# Supplementary material for: Identification and functional analysis of non-coding regulatory small RNA FenSr3 in Bacillus amyloliquefaciens LPB-18
Source: PeerJ. 2023 May 15;11:e15236. doi: 10.7717/peerj.15236 (PMC10194069; doi:10.7717/peerj.15236)
Supplement: Supplemental Information 4 [file peerj-11-15236-s004.zip › KO/CK-vs-T1_map/map04712.html]

KEGG PATHWAY: Circadian rhythm - plant - Reference pathway


|  |  |
| --- | --- |
| **Circadian rhythm - plant - Reference pathway** |  |

[
Pathway menu
| Organism menu
| Pathway entry
| Show description
| User data mapping
]

The circadian system is an endogenous rhythm with the period of about 24 hours to provide temporal organization of biological activity. In Arabidopsis the circadian oscillator at the core of this system is composed of the interlocked feedback loop formed by the major transcriptional factors CCA1 (circadian clock associated), LHY (late elongated hypocotyl) and TOC1 (timing of cab), a pseudo response regulator (PRR). The morning-expressed CCA1/LHY Myb transcription factors suppress TOC1 expression by binding to its promoter. Moreover, CCA1/LHY activates the expression of PRR7/9 in the morning and then PRR7/9 repress the transcription of CCA1/LHY during the rest of the day. By contrast, the evening-expressed TOC1 activates the expression of CCA1/LHY.

|  |  |  |
| --- | --- | --- |
| Reference pathway | 184% 150% 122% 100% 82% 67% 55% | 图片下载 |
